# Structurally Complex Osteosarcoma Genomes Exhibit Limited Heterogeneity within Individual Tumors and across Evolutionary Time

**DOI:** 10.1158/2767-9764.CRC-22-0348

**Published:** 2023-04-12

**Authors:** Sanjana Rajan, Simone Zaccaria, Matthew V. Cannon, Maren Cam, Amy C. Gross, Benjamin J. Raphael, Ryan D. Roberts

**Affiliations:** 1 Molecular, Cellular, and Developmental Biology Program, The Ohio State University, Columbus, Ohio.; 2 Center for Childhood Cancers and Blood Diseases, Abigail Wexner Research Institute at Nationwide Children's Hospital, Columbus, Ohio.; 3 Department of Computer Science, Princeton University, Princeton, New Jersey.; 4 Computational Cancer Genomics Research Group, University College London Cancer Institute, London, United Kingdom.; 5 Cancer Research UK Lung Cancer Centre of Excellence, University College London Cancer Institute, London, United Kingdom.; 6 Rutgers Cancer Institute of New Jersey, New Brunswick, New Jersey.; 7 The Ohio State University James Comprehensive Cancer Center, Columbus, Ohio.; 8 Division of Pediatric Hematology, Oncology, and BMT, Nationwide Children's Hospital, Columbus, Ohio.

## Abstract

Osteosarcoma is an aggressive malignancy characterized by high genomic complexity. Identification of few recurrent mutations in protein coding genes suggests that somatic copy-number aberrations (SCNA) are the genetic drivers of disease. Models around genomic instability conflict—it is unclear whether osteosarcomas result from pervasive ongoing clonal evolution with continuous optimization of the fitness landscape or an early catastrophic event followed by stable maintenance of an abnormal genome. We address this question by investigating SCNAs in >12,000 tumor cells obtained from human osteosarcomas using single-cell DNA sequencing, with a degree of precision and accuracy not possible when inferring single-cell states using bulk sequencing. Using the CHISEL algorithm, we inferred allele- and haplotype-specific SCNAs from this whole-genome single-cell DNA sequencing data. Surprisingly, despite extensive structural complexity, these tumors exhibit a high degree of cell-cell homogeneity with little subclonal diversification. Longitudinal analysis of patient samples obtained at distant therapeutic timepoints (diagnosis, relapse) demonstrated remarkable conservation of SCNA profiles over tumor evolution. Phylogenetic analysis suggests that the majority of SCNAs were acquired early in the oncogenic process, with relatively few structure-altering events arising in response to therapy or during adaptation to growth in metastatic tissues. These data further support the emerging hypothesis that early catastrophic events, rather than sustained genomic instability, give rise to structural complexity, which is then preserved over long periods of tumor developmental time.

**Significance:**

Chromosomally complex tumors are often described as genomically unstable. However, determining whether complexity arises from remote time-limited events that give rise to structural alterations or a progressive accumulation of structural events in persistently unstable tumors has implications for diagnosis, biomarker assessment, mechanisms of treatment resistance, and represents a conceptual advance in our understanding of intratumoral heterogeneity and tumor evolution.

## Introduction

Osteosarcoma is the most common primary bone tumor affecting children and adolescents (1). Nearly always high grade and aggressive, this disease exhibits extensive structural variation (SV) that results in a characteristically chaotic genome (2–4). With few recurrent point mutations in protein coding regions, osteosarcoma genomes often exhibit widespread structural complexity, giving rise to associated somatic copy-number aberrations (SCNA), a likely genomic driver of malignant transformation (5). Indeed, osteosarcoma is the prototype tumor whose study led to the discovery of chromothripsis (6, 7), a mutational process that causes the shattering of chromosomes leading to localized genomic rearrangements causing extreme chromosomal complexity (8). However, genomic complexity in osteosarcoma often goes beyond alterations caused by the canonical processes associated with chromothripsis (9, 10). Many have reasonably interpreted chromosomal complexity to be evidence of sustained chromosomal instability (CIN), often with supporting evidence from other cancer types (11–14). Indeed, cancer sequencing studies have identified the presence of extensive SCNAs as a marker for CIN (13).

Two distinct models have been proposed to explain the evolution of chromosomal structure and copy numbers in cancer genomes. One model suggests that underlying genomic instability gives rise to populations of cells with diverse phenotypic variations and that ongoing selection of advantageous phenotypes drives evolution and adaptation (15, 16). A somewhat competing model argues that discrete periods of genomic instability, isolated in tumor developmental time, give rise to extreme chromosomal complexity driven by a small number of impactful catastrophic events (17, 18). In osteosarcoma, investigators have put forward data that would seem to support both models. For instance, several groups have used single-cell RNA sequencing experiments, which reveal a high degree of transcriptional heterogeneity, to infer a high degree of copy-number heterogeneity within osteosarcoma tumors (19, 20), an observation which would support a malignant process driven by ongoing instability and gradual evolution. However, others have shown that SCNA profiles differ little when comparing primary with metastatic or diagnostic with relapse samples (5, 21, 22), which would suggest that ongoing mechanisms of malignancy do not create an environment of CIN^.^ Overall, it remains unclear whether the structurally complex genomes characteristic of osteosarcoma emerge from continuous cycles of diversification and fitness optimization within a context of ongoing instability and significant intratumoral chromosomal heterogeneity or from an early catastrophic event that gave rise to widespread structural changes that are then maintained over long periods of tumor development, with evidence from the literature supporting both potential mechanisms.

One challenge in addressing this question comes from challenges in data interpretation and deconvolution, as the existing studies describing copy-number clonality and evolution have inferred cell-specific copy-number states from bulk tumor sequencing, often from a single timepoint (23). However, investigating ongoing clonal evolution from bulk sequencing data remains particularly challenging, as each bulk tumor sample is an unknown mixture of millions of normal and cancer cells (24–27). The emergence of single-cell genomic DNA sequencing (DNA-seq) technologies now permits scalable and unbiased whole-genome single-cell DNA-seq of thousands of individual cells in parallel (24, 28), providing an ideal framework for analyzing intratumor genomic heterogeneity and SCNA evolution. Complementing these technical developments, recent computational advances—most notably the CHISEL algorithm (27)—enable highly accurate ploidy estimates and the inference of allele- and haplotype-specific SCNAs in individual cells and subpopulations of cells from low coverage single-cell DNA-seq. This allows cell-by-cell assessment of intratumoral SCNA heterogeneity, identification of allele-specific alterations and reconstruction of the evolutionary history of a tumor from thousands of individual cancer cells obtained at a single or multiple timepoints during tumor progression.

Here, we leverage these approaches to determine whether the widespread SCNAs in osteosarcoma result from ongoing genomic instability, providing a mechanism for tumor growth and evolution. Using expanded patient tissue samples, our studies revealed widespread aneuploidy and SCNAs in 12,019 osteosarcoma cells from 10 tumor samples. Using this approach, we found negligible intratumor genomic heterogeneity, with remarkably conserved SCNA profiles when comparing either the individual cells within a tumor or tumors collected at different therapeutic timepoints from the same patient. These findings suggest that the widespread patterns of genomic SVs in osteosarcoma are likely acquired early in tumorigenesis, and the resulting patterns of SVs and SCNAs can be preserved within an individual tumor, across treatment time and through the metastatic bottleneck.

## Materials and Methods

### Experimental Model—Expanded Patient Tissues and Murine Studies

#### Expanded Patient Tissue

Patient samples NCH-OS-4, NCH-OS-7, NCH-OS-8, NCH-OS-10, and NCH-OS-11 were obtained from patients who provided written informed consent under an Institutional Review Board (IRB)-approved protocol IRB11-00478 at Nationwide Children's Hospital (Human Subject Assurance Number 00002860). Germline whole-genome sequencing (WGS) was generated from patient blood collected under IRB-approved protocol IRB11-00478. Patient samples SJOS046149_X1, SJOS046149_X2, SJOS003939_X1, and SJOS031478_X2, with matched normal WGS were received from St. Jude's Children's Research Hospital, through the Childhood Solid Tumor Network (29–31). Written informed consent was obtained from all of these patients prior to tissue donation for research. The OS-17 patient-derived xenograft (PDX) was established from tissue obtained in a primary femur biopsy performed at St. Jude's Children's Research Hospital in Memphis and was a gift from Peter Houghton (32).

#### Tumor Fragment Expansion

Viable tissue fragments from patient tissue were expanded in C.B-17/IcrHsd-Prkdc^scid^ (RRID: IMSR_ENV:HSD-182) mice as subcutaneous tumors following approved Institutional Animal Care and Use Committee (IACUC) protocols. These tumors were allowed to grow to 300–600 mm^3^ before harvest. Passage 1 expanded tissue was used for all samples, with the exception of OS-17 (p18).

#### Orthotopic Primary Tumors

Single-cell suspensions of 5 × 10^5^ cells were injected intratibially in C.B-17/IcrHsd-Prkdc^scid^ mice as per IACUC guidelines. These tumors were harvested once they grew to 800 mm^3^ and prepped for single-cell DNA-seq.

### Single-cell Suspension and DNA Library Generation

Tumors harvested from mice were processed using the human tumor dissociation kit (Miltenyi Biotec, 130-095-929) on a GentleMacs Octo Dissociator with Heaters (Miltenyi Biotec, 130-096-427, RRID:SCR_020271). Single-cell suspensions in 0.04% BSA-PBS of dissociated tumor tissues were generated and frozen down using the 10X freezing protocol for SCNA. The frozen down single-cell suspensions were processed using the Chromium Single Cell DNA Library & Gel Bead Kit (10X genomics #1000040, RRID:SCR_019326) according to the manufacturer's protocol with a target capture of 1,000–2,000 cells. These barcoded single-cell DNA libraries were sequenced using the NovaSeq 6000 System (RRID:SCR_016387) using paired sequencing with a 100 bp (R1), 8 bp (i7), and 100 bp (R2) configuration and a sequencing coverage ranging from 0.01X to 0.05X (∼0.02X on average) per cell. Germline WGS was performed on NovaSeq SP 2 × 150BP.

### Single-cell SCNA Calling Using CHISEL

Paired-end reads were processed using the Cell Ranger DNA Pipeline (10X Genomics), obtaining a barcoded BAM file for every considered single-cell sequencing dataset. As described previously (27), the pipeline consists of barcode processing and sequencing-reads alignment to a reference genome, for which we used hg19. We applied CHISEL (v1.0.0) to analyze each generated barcoded BAM file using the default parameters and by increasing to 0.12 the expected error rate for clone identification in order to account for the lower sequencing coverage of the analyzed data (27). In addition, we provided CHISEL with the available matched-normal germline sample from each patient and phased germline SNPs according to the recommended pipeline by using Eagle2 (RRID:SCR_017262) through the Michigan Imputation Server with the Haplotype Reference Consortium reference panel (v.r1.1 2016). CHISEL inferred allele- and haplotype-specific copy numbers per cell and used these results to group cells into distinct tumor clones, while excluding outliers and likely noisy cells. To determine fraction of aberrant genome (genome affected by SCNAs), we defined aberrant as any nondiploid genomic region (i.e., allele-specific copy numbers different than {1, 1}) in tumors not affected by whole-genome doublings (WGD; NCH-OS-10, NCH-OS-4, and NCH-OS-7) or any nontetraploid genomic region (i.e., allele-specific copy numbers different than {2, 2}) in tumors affected by WGDs (NCH-OS-8, OS-17, NCH-OS-11, SJOS046149_X2, SJOS003939_X2, and SJOS003939_X1). We defined deletions as previously described in cancer evolutionary studies (26, 33–35). We say that a genomic region in a cell is affected by a deletion when any of the two allele-specific copy numbers inferred by CHISEL is lower than the expected allele-specific copy number (1 for non-WGD tumors or 2 for tumors affected by WGD). Conversely, a genomic region is amplified when any of the two allele-specific copy numbers is higher than expected.

### Reconstruction of Copy-number Trees

We reconstructed copy-number trees for tumor samples NCH-OS-4 (tibia), NCH-OS-7 (flank), and NCH-OS-7 (tibia), to describe the phylogenetic relationships between distinct tumor clones inferred by CHISEL based on SCNAs using the same procedure proposed in previous studies (27). Briefly, we reconstructed the trees using the maximum parsimony model of interval events for SCNAs (33, 34) and the copy-number profiles of each inferred clone. These copy-number profiles were obtained as the consensus across the inferred haplotype-specific copy numbers derived by CHISEL for all the cells in the same clone, where we also considered the occurrence of WGDs predicted by CHISEL. We classified copy-number events as deletions (i.e., del), as LOH which are deletions resulting in the complete loss of all copies of one allele (loh), as copy-neutral LOH which are LOHs in which the retained allele is simultaneously amplified, and as gains (gain).

### SCNA Calling on Whole-genome Data

To compare SCNA patterns across multiple tumor samples from the same patients, we downloaded a total of 47 whole-genome sequence datasets from St. Jude's DNAnexus (RRID:SCR_011884) from 14 patients including germline data and multiple tumor samples (diagnosis, relapse, metastasis, and xenograft). We also included the seven single-cell SCBA (scSCNA) datasets we generated which had matched germline whole-genome data in the St. Jude data and treated these as bulk sequencing data for this analysis. We used samtools (ref. 36; RRID:SCR_002105) to convert the bam files to fastq and aligned all datasets to a joint hg38/mm10 reference. We filtered out all mouse sequences and removed PCR duplicates. We then called SCNAs with Varscan (ref. 37; RRID:SCR_006849). Next, we combined all SCNA data by calculating the median copy number for 1,000 bp nonoverlapping bins. Correlation between samples was calculated using the cor function in R (RRID:SCR_001905) and the resulting output was plotted as a heatmap using the pheatmap R package (RRID:SCR_016418; https://github.com/raivokolde/pheatmap).

### SNP Calling on Whole-genome Data

To assess genetic heterogeneity of all samples, we produced phylogenetic trees from SNP data. We used the bam alignment files produced during the SCNA calling analysis and called SNPs using bcftools’ mpileup function (ref. 36; RRID:SCR_005227). We removed SNP calls with a quality below 20 and read depth below 20, and then generated vcf files using bcftools (36). To check TP53 status we merged the SNP calls with known SNPs from ClinVar (ref. 38; RRID:SCR_006169) and kept SNPs with a clinical significance (CLNSIG) of “Pathogenic” (39).

### Data and Code Availability

All the processed data, scripts, and results from CHISEL are available on GitHub (RRID:SCR_002630) at https://github.com/kidcancerlab/sc-OsteoCNAs. Patient-derived single-cell and whole-genome germline sequence data may be requested through dbGaP (accession: phs003209.v1) or St. Jude Cloud (29–31).

## Results

### Individual Cells within Osteosarcoma Tumors Exhibit Extensive SCNAs, but a High Degree of Homogeneity

Single-cell DNA-seq was performed on 12,019 tumor cells from expanded patient tissue samples. These nine patient tissues were obtained from diagnostic biopsies of localized primary tumors (*n* = 3), from postchemotherapy resection procedures (*n* = 2), or from relapsed metastatic lung lesions (*n* = 4), representing a spectrum of disease progression (Supplementary Tables S1 and S2). Apart from OS-17, a well-established model of metastatic osteosarcoma (32), all patient tissues were expanded for a single passage in mice as either subcutaneous flank or orthotopic bone tumors to obtain fresh tissue to perform single-cell DNA-seq (300–2,500 single cells per sample; Supplementary Fig. S1A). Previous studies have shown that this procedure yields samples with a high degree of fidelity relative to the diagnostic specimens, especially in early passages, an observation that we also validated in our own samples (40). We then used CHISEL (27) to identify allele- and haplotype-specific SCNAs from the sequencing data.

**FIGURE 1:**
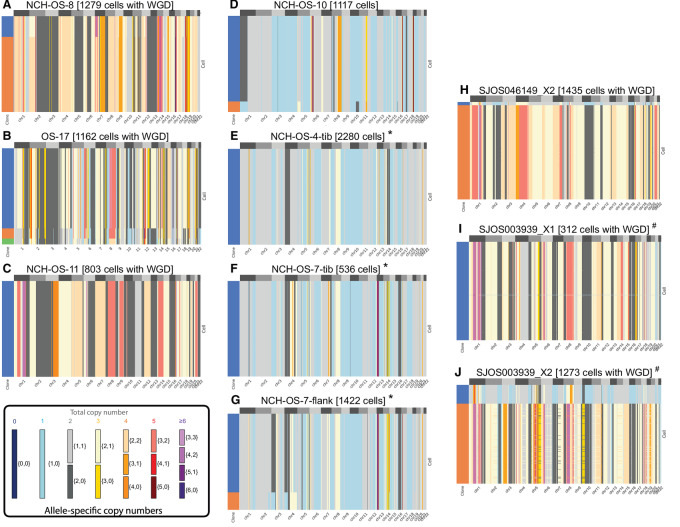
Extensive genomic complexity in 10 expanded osteosarcoma patient tissue samples using single-cell DNA-seq. **A–J**, Allele-specific copy numbers (heatmap colors) are inferred by using the CHISEL algorithm from each of 10 datasets including 300–2,300 single cancer cells from osteosarcoma tumors. In each dataset, cancer cells are grouped into clones (colors in leftmost column) by CHISEL based on the inferred allele-specific copy numbers. Corrected allele-specific copy numbers are correspondingly obtained by consensus. Note that cells classified as noisy by CHISEL have been excluded. “*” and “#” represent samples obtained from the same patient.

Consistent with previous reports (6, 41), sequencing showed a high degree of aneuploidy and extensive SCNAs across the entire osteosarcoma genome (Fig. 1). If driven by a process of CIN and ongoing/continuous clonal evolution, we would expect to observe multiple subclones with distinct complements of SCNAs within each same tumor, such as has been shown in recent single-cell studies of other cancer types (24, 27, 42). However, in each of the 10 samples investigated, we identified one dominant clone that comprised nearly all cells within each sample, with many samples composed entirely of a single clone (Supplementary Fig. S1B). To ensure that our results were not an artifact caused by the algorithm or the selected thresholds for noise control, we confirmed that the cells discarded as poor quality/noisy by CHISEL bear SCNAs similar to the dominant clones identified in each sample—thus no rare clones with distinct copy-number profiles were discarded inappropriately (Supplementary Fig. S2). Interestingly, we found that a substantial fraction of the overall copy-number changes involved allele-specific SCNAs, including copy-neutral LOHs (i.e., allele-specific copy numbers {2, 0}) that would have been missed by previous analyses of total copy numbers.

Genome-wide ploidy of single cells showed high variability across samples, ranging from 1.5 to 4, demonstrating a high degree of aneuploidy (Supplementary Fig. S3). Consistent with the high levels of aneuploidy, we identified the presence of WGD (a phenomenon identified with much greater precision in the single-cell data) across nearly all cancer cells of six tumors (NCH-OS-8, OS-17, NCH-OS-11, SJOS046149_X2, SJOS003939_X2, and SJOS003939_X1; Fig. 1A–C and H–J). One tumor (SJOS003939_X2) shows two subclones that appear to be undergoing whole-genome duplication, with one subclone exhibiting a SCNA pattern that is almost exactly double that of the other, across the genome.

To further assess tumor stability, we used paired datasets from patients collected at timepoints along tumor progression. We observed that whole-genome copy-number profiles were highly consistent within each patient. The first set includes NCH-OS-4, which was obtained shortly after diagnosis at the time of resection (after two rounds of neoadjuvant chemotherapy with methotrexate, doxorubicin, and cisplatin), and NCH-OS-7, which was obtained at the time of relapse the following year. Comparing genomic windows where at least one sample had a SCNA in the primary clone, 77%–78% of genomic windows had identical copy-number assignments in both samples, despite variation in tumor purity (Supplementary Fig. S4). This contrasts with between 1% and 35% concordance for nonrelated samples. The correlation between related samples may be even higher, given inaccuracies expected from low-coverage scSCNA detection.

The second set of paired primary and metastatic lesions (SJOS003939_X1, SJOS003939_X2) also showed SCNA profiles that were highly similar (58% of SCNAs identical; Supplementary Fig. S4), suggesting a high degree of conservation of genomic aberration profiles over therapeutic time. Overall, we observed a very high degree of homogeneity within cancer cells sequenced from the same tumor. Even in tumors where small proportions of cells (5%–20%) are classified as part of small subclones, these subclonal cells are only distinguished by modest SCNAs differences within a few chromosomes. The exception to this general observation arose in SJ0S003939_X2, a second xenograft from a patient with a germline TP53, raising suspicion for a second malignancy (rather than a relapse). Thus, despite the high levels of aneuploidy and massive SCNAs identified in all 10 samples, these osteosarcoma cells demonstrated very modest levels of intratumor heterogeneity and variation across therapeutic time.

### Osteosarcoma Cells Harbor Extensive SCNAs That Mostly Correspond to Deletions

**FIGURE 2:**
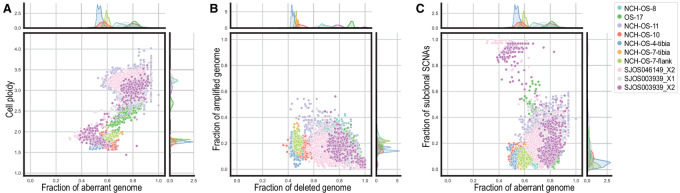
Osteosarcoma cancer cells exhibit extensive genetic alterations, especially deletions, but a relatively low level of heterogeneity. **A,** Ploidy (*y*-axis) and fraction of aberrant genome (*x*-axis) of every cell (point) across the 10 analyzed datasets (colors). The kernel density of the marginal distributions of each value is reported accordingly in every plot. **B,** Fraction of genome affected by deletions (*x*-axis) versus fraction of genome affected by amplifications (*y*-axis) of every cell (point) across the 10 analyzed datasets (colors). **C,** Fraction of aberrant genome (*x*-axis) and fraction of subclonal SCNAs (i.e., fraction of the genome with SCNAs different than the most common clone for the same region across all cells in the same dataset, *y*-axis) of every cell (point) across the 10 analyzed datasets (colors).

The occurrence of WGD events correlates with high levels of aneuploidy and higher frequency of SCNAs (43). Recent reports have identified that WGDs serve as a compensatory mechanism for cells to mitigate the effects of deletions (44). We investigated cell ploidy and fraction of genome affected by SCNAs (aberrant), amplifications, deletions, and subclonal CNAs between tumors affected by WGDs (NCH-OS-8, OS-17, NCH-OS-11, SJOS046149_X2, SJOS003939_X2, and SJOS003939_X1) and tumors not affected by WGDs (NCH-OS-10, NCH-OS-4, and NCH-OS-7). Osteosarcoma cells in all analyzed tumors demonstrate extensive SCNAs, affecting more than half of the genome in every tumor cell. We found that the fraction of genome affected by SCNAs ranged from 50% to 70% on average (Fig. 2A; Supplementary Fig. S5A). This result might not be surprising for tumors affected by WGDs; however, we observed that tumors not affected by WGD had a high fraction of aberrant genome as well (higher than 50% on average; Fig. 2A). This aberrant fraction is substantially higher than has been reported for other cancer types (43).

We observed a clear enrichment of deletions among the identified SCNAs across all cancer cells. The fraction of genome affected by amplifications is 0%–40% on average in every tumor, while the fraction of the genome affected by deletions is 40%–100% on average across all cancer cells in every tumor (Fig. 2B). This result is not particularly surprising for tumors with WGD events and is consistent with a recent study of López and colleagues (44) that demonstrated a similar correlation in patients with non–small cell lung cancer. However, in the osteosarcoma tumors analyzed in this study, we found that cancer cells in non-WGD tumors are similarly affected by a high fraction of deletions (Fig. 2B). Importantly, we observed that >80% of the cells in all but two of our samples harbored LOH events at the TP53 locus [in-line with frequency reported previously (3)] (Supplementary Fig. S6). This substantiates the correlation between LOH of TP53 and high levels of genomic instability (including the occurrence of WGDs) reported in recent studies (13, 44, 45), and suggests that these events might have a critical role in the maintenance of a highly aberrant genomic state. Notably, CHISEL identified 50% of the samples to harbor copy-neutral LOH alterations at the TP53 locus that would be missed by total copy-number analyses (Supplementary Fig. S6).

We found it interesting that subclonal SCNAs that likely occurred late in the evolutionary process (present only in subpopulations of cancer cells) are relatively rare across all analyzed osteosarcoma tumors, irrespective of WGD status (with a frequency of 0%–20% in most cancer cells; Fig. 2C; Supplementary Fig. S5). Note the only exceptions to this observation correspond to cells in NCH-OS-11, a sample with overall higher noise and variance, and a subpopulation of cells in two other tumors (SJOS046149_X2 and SJOS003939_X2) that appear to be cells that have not undergone WGD (Supplementary Fig. S5E). Indeed, the average fraction of SCNAs in SJOS046149_X2, SJOS003939_X2 is lower than 20%. Overall, we observed that osteosarcoma cells investigated in these 10 samples, whether passaged in cell culture over a few generations (OS-17), treatment naïve or exposed to extensive chemotherapy, bear high levels of aneuploidy marked with extensive deletions and negligible subclonal diversification, irrespective of WGD status.

### Longitudinal Single-cell Sequencing Shows Modest Evolution of SCNA from Diagnosis to Relapse

Increased aneuploidy has previously been associated with CIN and accelerated tumor evolution (13, 46), though some have suggested that this observation specifically applies to tumors that exhibit not only high levels of SCNA, but also high levels of subclonal SCNA (47). To assess the degree of structural instability exhibited by these tumors, we examined a pair of samples, NCH-OS-4 and NH-OS-7, collected at diagnosis and at relapse respectively, from the same patient to determine whether SCNAs remained stable over therapeutic time or showed signs of significant instability/evolution. This included an expansion in both the flank and orthotopic tibia locations to determine whether these environments drove a niche-specific expansion of a selected clone. Results suggest that expansion in mouse did not lead to evolutionary disequilibrium.

**FIGURE 3:**
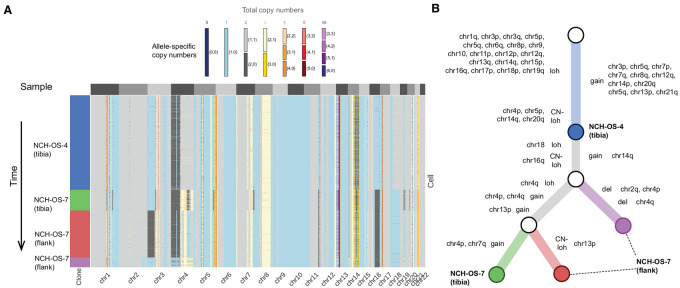
Phylogenetic reconstruction of tumor evolution is consistent with longitudinal ordering of matched tumor samples and reveals conservation of SCNA profiles. **A,** Allele-specific copy numbers (heatmap colors) across all autosomes (columns) have been inferred by CHISEL jointly across 4,238 cells (rows) in three tumor samples from the same patient: one pretreatment sample (NCH-OS-4 tibia) and two posttreatment samples (NCH-OS-7 tibia and NCH-OS-7 flank). CHISEL groups cells into four distinct clones (blue, green, red, and purple) characterized by different complements of SCNAs. **B,** Phylogenetic tree describes the evolution in terms of SCNAs for the four identified tumor clones. The tree is rooted in normal diploid clone (white root) and is characterized by two unobserved ancestors (white internal nodes). Edges are labeled with the corresponding copy-number events that occurred and transformed the copy-number profile of the parent into the profile of the progeny. The four tumor clones (blue, green, red, and purple) are labeled according to the sample in which they were identified.

We used CHISEL to jointly analyze 4,238 cells from these paired tumor samples and to infer corresponding allele- and haplotype-specific SCNAs (Fig. 3A). On the basis of existing evolutionary models for SCNAs, we reconstructed a phylogenetic tree that describes the evolutionary history of the different tumor clones identified in these tumors (Fig. 3B). The result from this phylogenetic analysis confirmed our findings in two ways. First, we found that the evolutionary ordering of the different clones in the phylogenetic tree is concordant with the longitudinal ordering of the corresponding samples (Fig. 3B): the tumor clones identified in the early sample (NCH-OS-4) correspond to ancestors of all the other tumor clones identified in later samples (NCH-OS-7-tib and NCH-OS-7-flank). Second, we observed that the vast majority of SCNAs accumulated during tumor evolution are truncal, indicating that these aberrations are accumulated early during tumor evolution and shared across all the extant cancer cells (Fig. 3B). In fact, only three significant events distinguish the most common ancestor of all cells from this patient (identified in NCH-OS-4) from the cells within the relapse lesion: gain of chromosome 14, gain of chromosome 16q (resulting in copy-neutral LOH), and deletion of one allele of chromosome 18 (resulting in LOH). Note that we cannot be certain of when these clones arose. It is possible these changes occurred early in tumor formation and were present in the primary tumor but were not present in the biopsied sample and so we must exercise caution when assessing whether there is ongoing low-level CIN.

To further assess the effects that environmental stressors might play on the creation and/or emergence of subdominant clones, which could be masked because of extreme rarity, we expanded samples from the same tumor within two different microenvironments in mice. Consistent with the diagnosis-relapse sample comparison, clones identified within tumors grown orthotopically within the tibia (NCH-OS-7-tib) or within subcutaneous flank tissues (NCH-OS-7-flank) are highly concordant (78% of genomic windows called with identical SCNA values) and distinguished by few focal SCNAs (primarily single copy changes). These changes could be either be variance in SCNA calling from the sequencing data, stochastic differences caused by the presence of subclones within the original tumor sample that was bisected and implanted or biologically relevant. Without targeted studies, it is not possible to confidently define the biological role of these focal changes, if any. A third comparison of tumors separated in time and space was possible using another paired set of primary and metastatic lesions (SJOS003939_X1, SJOS003939_X2), which also demonstrated negligible subclonal diversification (Supplementary Fig. S7). Indeed, each sample was dominated by one major clone, which exhibited only subtle differences from the paired sample. While these results do not suggest the absence of SCNA changes over the course of tumor evolution, they do suggest a level of stability quite similar to genomically simple tumors and that the mechanisms giving rise to these limited focal changes are different from those that gave rise to widespread genomic complexity.

**FIGURE 4:**
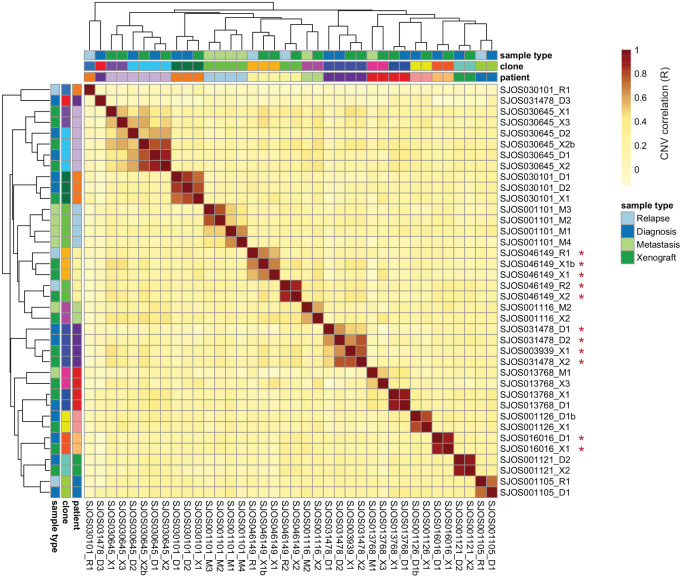
CNA correlation between osteosarcoma samples. Pearson *R* values denoting correlation of binned copy numbers between samples. Colors on *x* and *y*-axes indicate each sample's patient of origin and type as well as the clones defined from the correlation analysis. Red asterisks denote samples from patients with germline TP53 mutations. Note that SJOS003939_X1 is from the same patient as SJOS031478_* samples.

To further explore temporal and spatial consistency of patient tumor samples, we combined WGS data obtained from paired osteosarcoma samples within the St. Jude database (29–31) with our own WGS and performed SCNA analysis. This combined data yielded between two and six tumor samples for each patient, in addition to a germline reference sample. In most cases, all samples taken from a single patient at different timepoints were highly similar and clustered together (Fig. 4; Supplementary Fig. S8A–K). There were, however, five samples that had more than one distinct clone in separately collected samples which reduced the overall average. In these instances, the average correlation between clonal populations within a patient was only 0.28, which was close to the correlation we observed between samples taken from different patients (mean Pearson correlation = 0.18). Deeper exploration of these samples revealed germline TP53 mutations in some patients (shown with a red asterisk in Fig. 4), suggesting an underlying cancer predisposition and a likelihood that these are tumors arising from distinct oncogenic events. The correlation within a clone was very high (mean Pearson correlation = 0.67), despite the noise created by the sparse coverage sequencing inherent to this method. Xenograft-derived samples did not cluster separately from samples derived directly from patients (Fig. 4), except for two samples from SJOS030645 which formed a distinct cluster. The xenograft samples had a high correlation with non-xenograft samples from the same patient (mean Pearson correlation = 0.70), suggesting that the SCNA patterns in these samples were not dramatically altered by clonal selection within the mouse. Determination of SCNA-based clonal composition and tumor purity was performed using the HATCHet algorithm (26), providing additional context for interpretation of results. HATCHet results show a very high degree of SCNA-based clonal conservation from one clinical timepoint to the next.

## Discussion

Osteosarcoma is one of several malignancies typified by chaotic genomic landscapes dominated by SV and corresponding copy-number alterations (3). Chromosomal complexity in osteosarcoma and other cancers with complex karyotypes has often been assumed to represent underlying genomic instability, suggesting that these tumors gradually accumulate structural changes that lead to increasing complexity, with continual selection of ever more aggressive clones driving malignant progression. This concept was supported by previous reports demonstrating that, in some patients, spatially separated tumor samples exhibit slightly divergent SNP and SCNA patterns (48, 49). These studies also noted that, while there is heterogeneity between samples, there seem to be clones that are shared across multiple metastatic foci. By nature, these studies understandably focused on identifying SNP and copy-number differences contained within distinct lesions in these highly aggressive tumors, with the largest sample sets collected at autopsy. By utilizing single-cell DNA-seq, we have been able to investigate intratumor genomic heterogeneity and tumor evolution in concrete ways that were previously possible only by estimation and inference using bulk sequencing methods (50–53). This allowed us to ask different questions related to the stability of these complex genomes within a tumor sample. We were surprised to find that cells within a tumor demonstrated surprisingly little cell-to-cell variability in SCNA profiles—results that, at first, seemed discordant with previous reports of intratumoral genomic heterogeneity in osteosarcoma (19).

Analyzing longitudinal sets of paired samples, we showed that these particular osteosarcoma tumors maintained relatively stable SCNA profiles from diagnosis to relapse, primary tumor to metastasis, and during growth in two distinct environments. Our phylogenetic analysis suggested that the most recent common ancestor of these related samples harbored almost all of the observed SCNAs, suggesting that most of the genomic aberrations arose early in the tumorigenic process within these patients, followed by a long period of stable clonal expansion (clonal stasis; ref. 18). Our analysis of bulk whole-genome sequence data from St. Jude supports this observation and highlights an important observation. Where we observed multiple clones in our single-cell data, each clone was homogeneous in its SCNA patterns across cells within the clone, but highly distinct from other clones (Fig. 1). We observed the same phenomenon in the bulk St. Jude data where a single clone detected in multiple temporally or anatomically separated samples had highly conserved SCNAs while distinct clones were highly divergent (Fig. 4). This suggests that each of these clones either derive from a very early event that produced multiple distinct clones, or independent tumorigenesis events.

An inherent limitation of single-cell analysis of biopsy samples is that they are not representative of the entire tumor and so the homogeneous cell populations we observe could, in part, derive from the small sample size involved. However, our data include independent data from multiple biopsies that showed similar clonal patterns. Also, our analysis of the St. Jude samples includes multiple independent biopsies from patients and demonstrates the same pattern of SCNA conservation across samples’ single cell. A potential unexpected advantage of the small sample size inherent to tumor biopsies is that these samples tended to be clonal in nature in our single-cell data. Given this, it may be feasible to assume that bulk SCNA results are representative of most cells within the biopsy.

Another limitation of biopsy samples is the potential for normal cell types within the sample to interfere with the evaluation of SCNAs. If too large of a proportion of normal cells are present, estimates of copy number will be less accurate. For instance, Supplementary Fig. S8A shows sample SJOS031478_D1 which has very low copy-number alteration values, suggesting that this sample may have a large proportion of normal cells, making detection of SCNA difficult. Deconvolution can improve, but not fully overcome, this issue (26). To help compensate for this issue, for Fig. 4 we used correlation between samples instead of comparison of absolute copy numbers. This allows sample SJOS031478_D1 to cluster closely with SJOS031478_D2 in Fig. 4 despite apparent contamination with normal tissue. Samples SJOS031478_D1 and SJOS031478_D3 had very low correlation in their SCNA patterns. It is notable that these samples harbor distinct SCNAs including a deletion of a large portion of chromosome five in SJOS031478_D3 that is absent from SJOS031478_D1 and a large amplification of chromosome eight present in SJOS031478_D1 but absent from SJOS031478_D3 (Supplementary Fig. S8A) indicating that these are distinct clones. Similar patterns can be seen in Supplementary Fig. S8J where SJOS046149_R2 and SJOS046149_X2 are distinct from SJOS046149_R1, SJOS046149_X1 and SJOS046149_X1b.

One genomic change that was readily evident within our data was the common occurrence of WGD. Using the CHISEL algorithm (27), we identified high levels of aneuploidy and extensive genomic aberrations that were dominated by deletions within these osteosarcoma tumors. Consistent with previous reports suggesting WGD as a mechanism to mitigate the effects of widespread deletions (44), we identified extensive deletions even in tumors that had not undergone duplication. Indeed, some of our samples showed subclones of cells that differed across the genome by almost exactly 2-fold, which may represent populations of cells that had undergone duplication (with the duplicated fraction being the dominant clone). These findings support the hypothesis that duplication is a process that produces a more aggressive clone from cells that are first affected by widespread deletion.

To expand the analysis addressing the question of stability beyond our single cell WGS samples, we evaluated SNCAs in bulk WGS data derived from osteosarcoma samples. We investigated paired tumor samples across 14 patients (and included the associated PDXs, where available) to determine whether SCNA patterns were stable across time. We observed that there were both identical and divergent clones within single patients. Clones were similar with correlations as high as 0.92. In the few patients where relapse specimens contained highly divergent clones (Supplementary Fig. S8A–K), a deeper analysis revealed germline TP53 mutations in many cases (Fig. 4). In these patients harboring a genetic predisposition to developing osteosarcoma, it is likely that these genetically distinct lesions represent independent oncogenic events and it is possible that TP53 activity was impaired through alternative means in the other patients.

Historically, studies in osteosarcoma (and other cancers) have equated a high level of SCNA with ongoing genomic instability (41, 54), and some direct evidence has supported this concept (49). However, several recent studies seem to challenge this conclusion, showing preservation of SCNA profiles in primary versus metastatic and diagnostic versus relapse samples (5, 22, 48). Our findings support the hypothesis that mechanisms leading to widespread structural alterations are active early in tumorigenesis but resolve and are followed by long periods of relative stability. These seemingly discordant observations may both be true. First, there may be different paths to chromosomal complexity in different tumors—processes that resolve in some tumors, but do not in others. Indeed, nearly all these publications contain sample sets that seem to support higher and lower levels of chromosomal (in)stability.

Second, tumor cells may experience time-limited periods of relative instability, resulting in the phenomenon of punctuated equilibrium, as has been shown in other cancer types (18). In a punctuated equilibrium scenario, the timing of the biopsy would change the likelihood of finding more or less SCNA heterogeneity within the tumor using methods like single-cell WGS.

**FIGURE 5:**
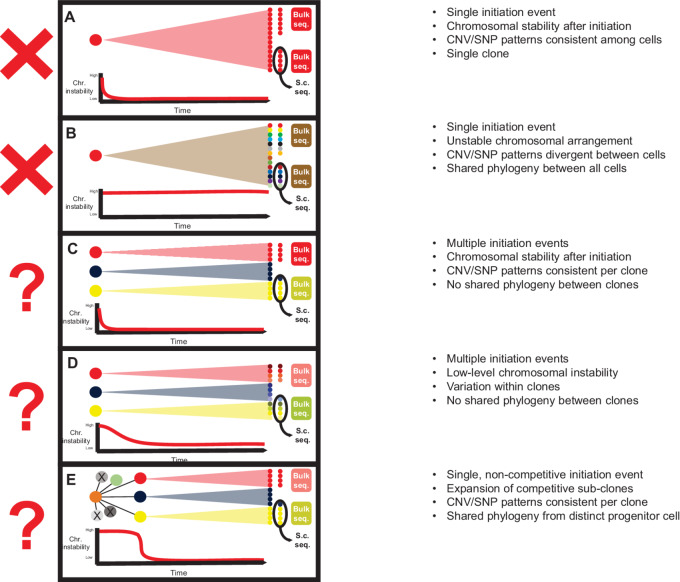
Possible models for temporal SCNA stability. **A,** After tumor initiation, if CIN is low, tumors will have identical SCNA patterns across all cells. **B,** High tumor instability would result in tumors with highly heterogeneous SCNA patterns across cells which may not be apparent in bulk sequencing. **C,** If there are multiple initiation events with low subsequent genomic instability SCNA patterns will be consistent across clones, which will be apparent by both bulk and single-cell sequencing. **D,** If there are multiple initiation events with ongoing genomic instability, clones derived from each will be similar but with highly variable SCNA patterns within a clone. This heterogeneity would be apparent by single-cell sequencing but not bulk sequencing. **E,** If a single initiation event is followed by an initial period of genomic instability, divergent clones could emerge. Patterns of stability within a clone would suggest that chromosomal stability is reestablished prior to clonal expansion.

To synthesize these concepts, there are several potential models for the emergence of SCNA-defined clones in osteosarcoma (Fig. 5). A single initiation event giving rise to a single dominant clone followed by highly stable genomic organization (Fig. 5A) would cause all tumor samples from a patient to have consistent SCNA patterns in both bulk and single-cell sequencing. This mechanism, however, would not be supported by previously published data (48, 49) or the results presented here.

In an alternative model, instability persists from the oncogenic insult forward (Fig. 5B). This model would produce samples containing multiple divergent subclones, which would be evident in bulk sequencing collected in different loci, though not detectable within a single sample. Single-cell analyses would identify several SCNA-defined clones within each sample. This model seems less likely considering our single-cell results.

A third outcome could result if there were multiple independent initiation events, producing several competing clones within a tumor, followed by a period of stable chromosomal organization (Fig. 5C). This mechanism, which is consistent with the punctuated equilibrium hypothesis (18), could produce multiple samples from a patient with divergent SCNA patterns by bulk sequencing; however, cells within each sample would demonstrate highly similar copy-number patterns (assuming the sample does not overlap a boundary between clones). This model agrees with both published observations (48, 49) and the results presented here.

A slight modification of this model would invoke early mechanisms giving rise to multiple competing clones, followed by a period where an independent mechanism causes ongoing low-level CIN within each founder clone (Fig. 5D). This mechanism would generate multiple slightly divergent clones within each sample. We see some evidence to support this model, such as the similar, but distinct, clones observed in the NCH-OS-7 samples in Figs. 1 and 3. These patterns could also be explained by tissue sampling bias, experimental variance or computational noise caused by the low sequencing depth inherent to single-cell data. A larger study would be needed to evaluate this.

A final model, which would also be consistent with both our single-cell data and the published record, suggests a single initiation event followed by a period of where daughter cells exhibit CIN (Fig. 5E), creating a diversity of competing clones. Eventually, clones emerge that exhibit chromosomal stability and have a competitive advantage. In this scenario, tumor cells within a patient are distinct from clone to clone, but homogeneous within a clone, with a small subset of shared SCNAs that were present in the origin cell and maintained through the subsequent CIN.

While copy-number patterns might be stable after an initial structure-altering event, single-nucleotide variants (SNV) arise through completely different mechanisms and likely have different evolutionary dynamics. Previous studies looking at osteosarcoma across therapeutic time show clear sequence related changes dominated by patterns that suggest a cisplatin-induced mutational burden (49). However, the structural integrity of the chromosomes does not seem to be affected by treatment (48, 49).

A brief (single cycle) expansion of tissue using an animal host proved useful for generating high-quality single-cell suspensions of sufficient quantity while maintaining high fidelity to the original patient sample. This approach is not intended to model tumor progression in a murine host, but rather to maximize the data obtained from each of these incredibly valuable samples. Some have expressed concerns that mouse-specific evolution selects for subclonal populations (55). However, the mouse-specific evolution that occurs over many passages (such as in the development of a PDX) does not occur when the mouse is used as a vehicle for brief expansion (40). Our SCNA analysis comparing results of these expanded tissues to bulk sequencing performed directly on the patient samples showed a very high correlation between expanded and primary samples. Therefore, this approach may represent a productive compromise enabling multiple lines of research on tissues with limited availability in rare diseases.

Our findings of clonal stasis in osteosarcoma sheds some light on the complex evolutionary history of this cancer type and could have important implications for tumor evolution, patient diagnosis and treatment of osteosarcoma. However, a much larger sample size of patient tissues is needed to capture the full heterogeneity of osteosarcoma seen in the human disease and describe the prevalence of multiple tumor subclones. Somewhat ironically, one may conclude from this data that bulk sequencing methods likely produce an adequate assessment of SCNA profiles and heterogeneity in osteosarcoma, given the lack of heterogeneity found in our analysis. These data likewise suggest that, in a clinical setting, sequencing analyses based on SCNA likely remain valid, even into treatment and relapse, assuming separate samples derive from the same clonal tumor population.

At a biological level, these results support the early catastrophe model as a primary mechanism of osteosarcoma complexity, suggesting that most structural rearrangements occur early in the tumorigenic process. While other rearrangements certainly can occur during malignant progression, subsequent structural events do not appear to be necessary for invasion, metastasis, or therapeutic resistance (though they certainly may contribute to such processes), nor do they appear to be the same mechanisms that create widespread structural complexity. Ongoing research will continue to inform our understanding of the contributions that initial catastrophic events and ongoing mechanisms of genomic evolution have and how they influence clinical outcomes.

It is important to note that our study is performed in a way that is generally insensitive to other alterations (such as SNVs) as a source of genomic variation, though few recurrent mutations have been identified in osteosarcoma, despite extensive genetic analysis (5, 54, 56). If both observations hold true, one must conclude that the acquisition of traits that drive malignant progression arise through epigenetic-based evolutionary processes, which remain poorly understood. Interestingly, we and others have shown that these same osteosarcomas demonstrate a high level of intratumor transcriptional heterogeneity (19, 57). This heterogeneity of gene expression in cells that are genomically homogeneous suggests that there may be microenvironmental differences or an underlying epigenetic heterogeneity, which could be a basis for competition and selection of tumor cells.

## Supplementary Material

Supplementary Table S1 Characteristics of patient samples used in this study

Supplementary Figure S1 Cell count and fraction of clones

Supplementary Table S2 Characteristics of patient samples obtained from Childhood Solid Tumor Network

Supplementary Figure S2 CHISEL output plots

Supplementary Figure S3 Ploidy plots

Supplementary Figure S4 Proportion of identically assigned SCNAs between samples

Supplementary Figure S5 Fraction of altered genome density plots

Supplementary Figure S6 TP53 copy number in single-cell data

Supplementary Figure S7 CHISEL plot for SJOS003939 samples

Supplementary Figure S8 Bulk data genome SCNA plots by patient
