# Supplementary material for: Structurally Complex Osteosarcoma Genomes Exhibit Limited Heterogeneity within Individual Tumors and across Evolutionary Time
Source: Cancer Res Commun. 2023 Apr 12;3(4):564–75. doi: 10.1158/2767-9764.CRC-22-0348 (PMC10093779; doi:10.1158/2767-9764.CRC-22-0348)
Supplement: Supplementary Figure S1 — Cell count and fraction of clones [file crc-22-0348-s02.pdf]

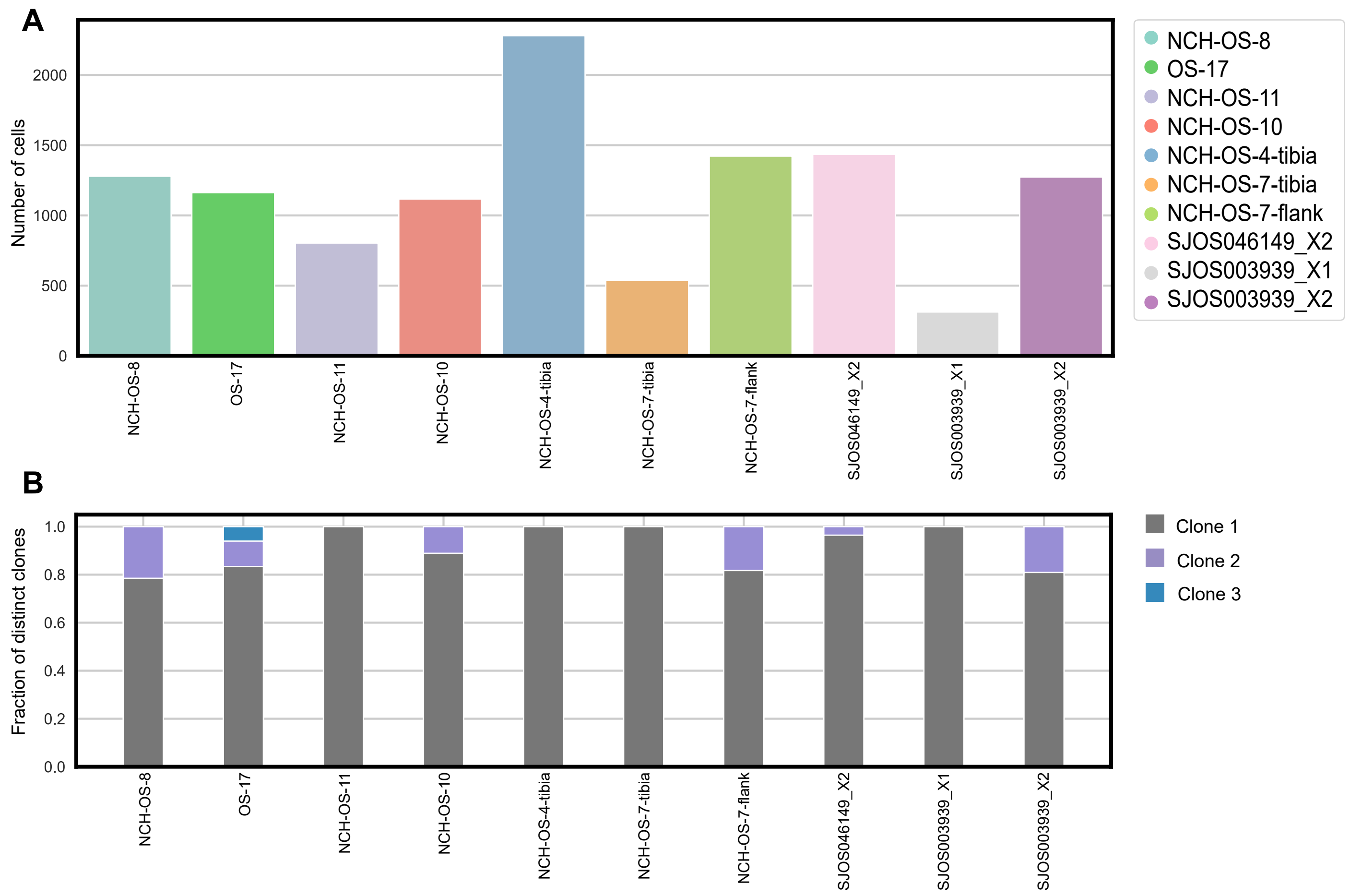

Supplemental Figure S1: Cell count and fraction of clones. A. Quantification of cells captured for single cell DNA sequencing in each of the samples. B. Stacked barplots showing the composition of each sample by clone as determined by CHISEL.
