# Supplementary material for: Structurally Complex Osteosarcoma Genomes Exhibit Limited Heterogeneity within Individual Tumors and across Evolutionary Time
Source: Cancer Res Commun. 2023 Apr 12;3(4):564–75. doi: 10.1158/2767-9764.CRC-22-0348 (PMC10093779; doi:10.1158/2767-9764.CRC-22-0348)
Supplement: Supplementary Figure S2 — CHISEL output plots [file crc-22-0348-s04.pdf]

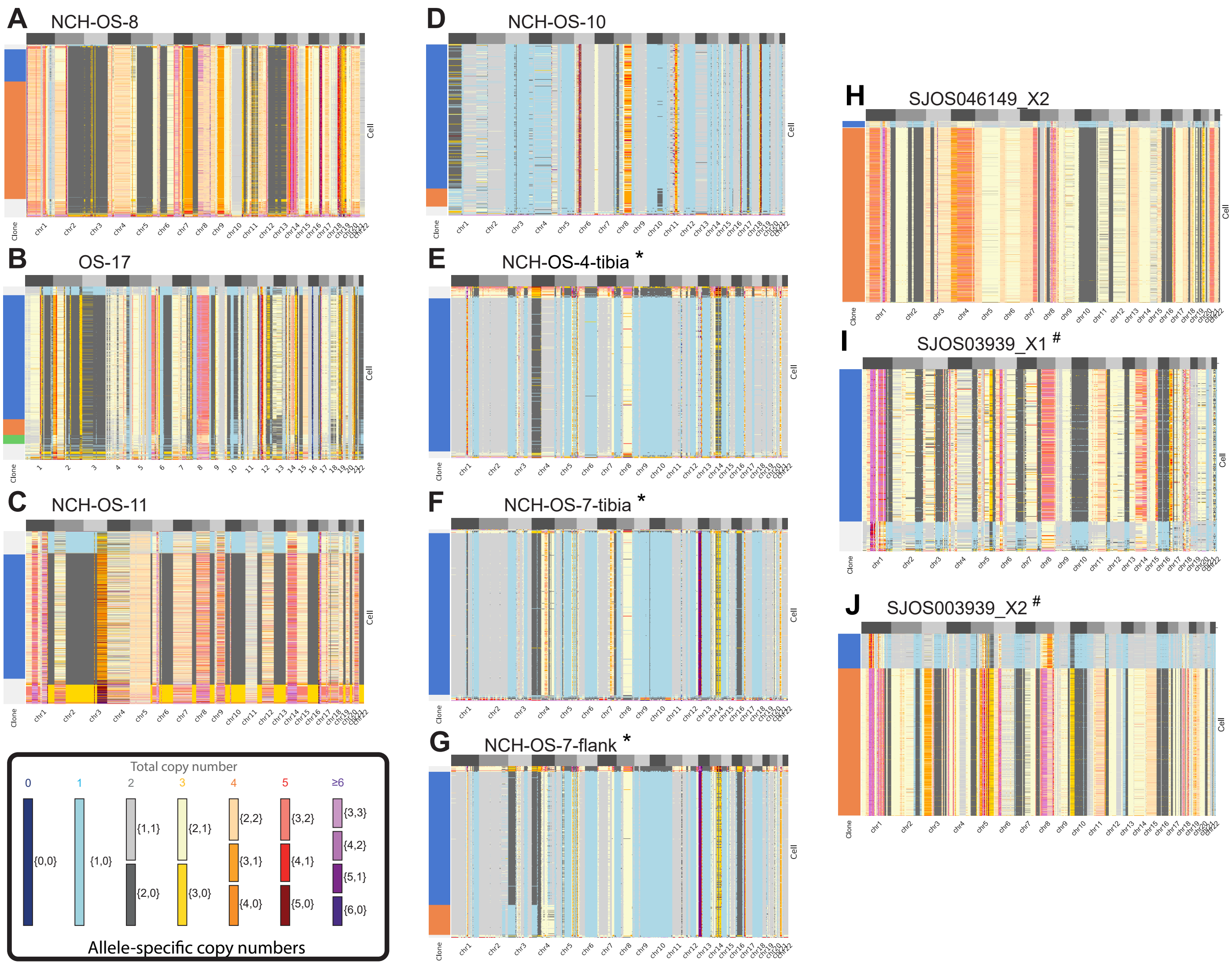

Supplemental Figure S2: CHISEL output plots. A-J. Raw, unfiltered copy number profiles (without filtering noisy cells) for the same data shown in Figure 1.
