# Supplementary material for: Structurally Complex Osteosarcoma Genomes Exhibit Limited Heterogeneity within Individual Tumors and across Evolutionary Time
Source: Cancer Res Commun. 2023 Apr 12;3(4):564–75. doi: 10.1158/2767-9764.CRC-22-0348 (PMC10093779; doi:10.1158/2767-9764.CRC-22-0348)
Supplement: Supplementary Figure S4 — Proportion of identically assigned SCNAs between samples [file crc-22-0348-s06.pdf]

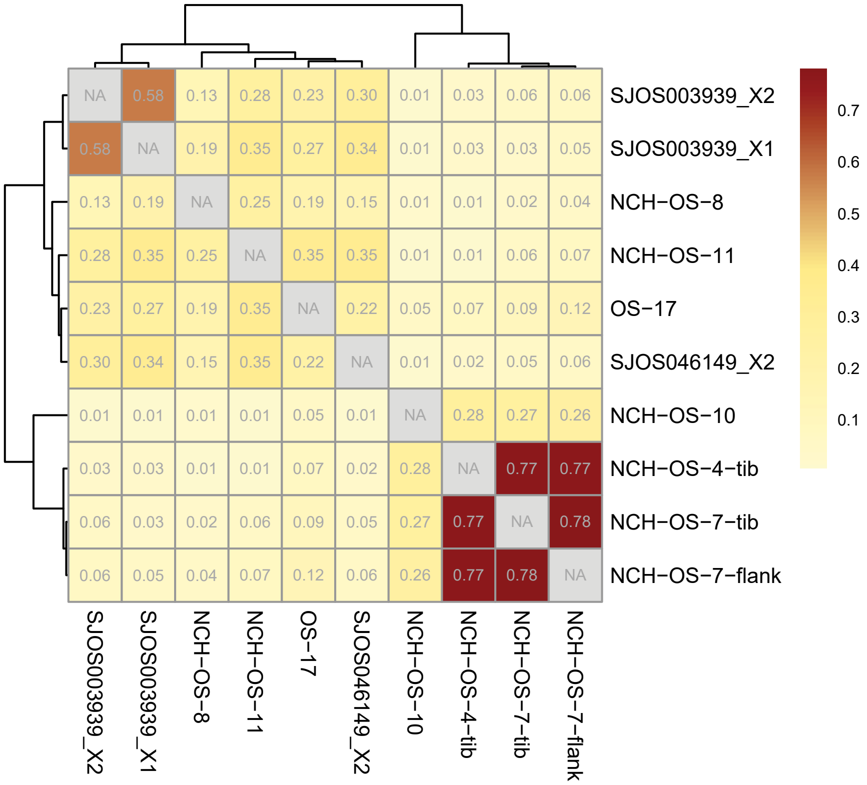

Supplemental Figure S4: Proportion of identically assigned SCNAs between samples. Heatmap showing the proportion of genome showing identical copy numbers between samples using the genomic windows that were called by CHISEL. This excludes windows where both samples were copy number neutral to avoid artificial inflation of values in samples with few SCNAs. Note that these results are, by nature, a very conservative quantification of the minimal degree of pairwise shared identity due to the inherent noise and variability within the data (and should be considered an “at least” value) but are useful for comparing similarity among groups of samples.
