# Supplementary material for: Structurally Complex Osteosarcoma Genomes Exhibit Limited Heterogeneity within Individual Tumors and across Evolutionary Time
Source: Cancer Res Commun. 2023 Apr 12;3(4):564–75. doi: 10.1158/2767-9764.CRC-22-0348 (PMC10093779; doi:10.1158/2767-9764.CRC-22-0348)
Supplement: Supplementary Figure S5 — Fraction of altered genome density plots [file crc-22-0348-s07.pdf]

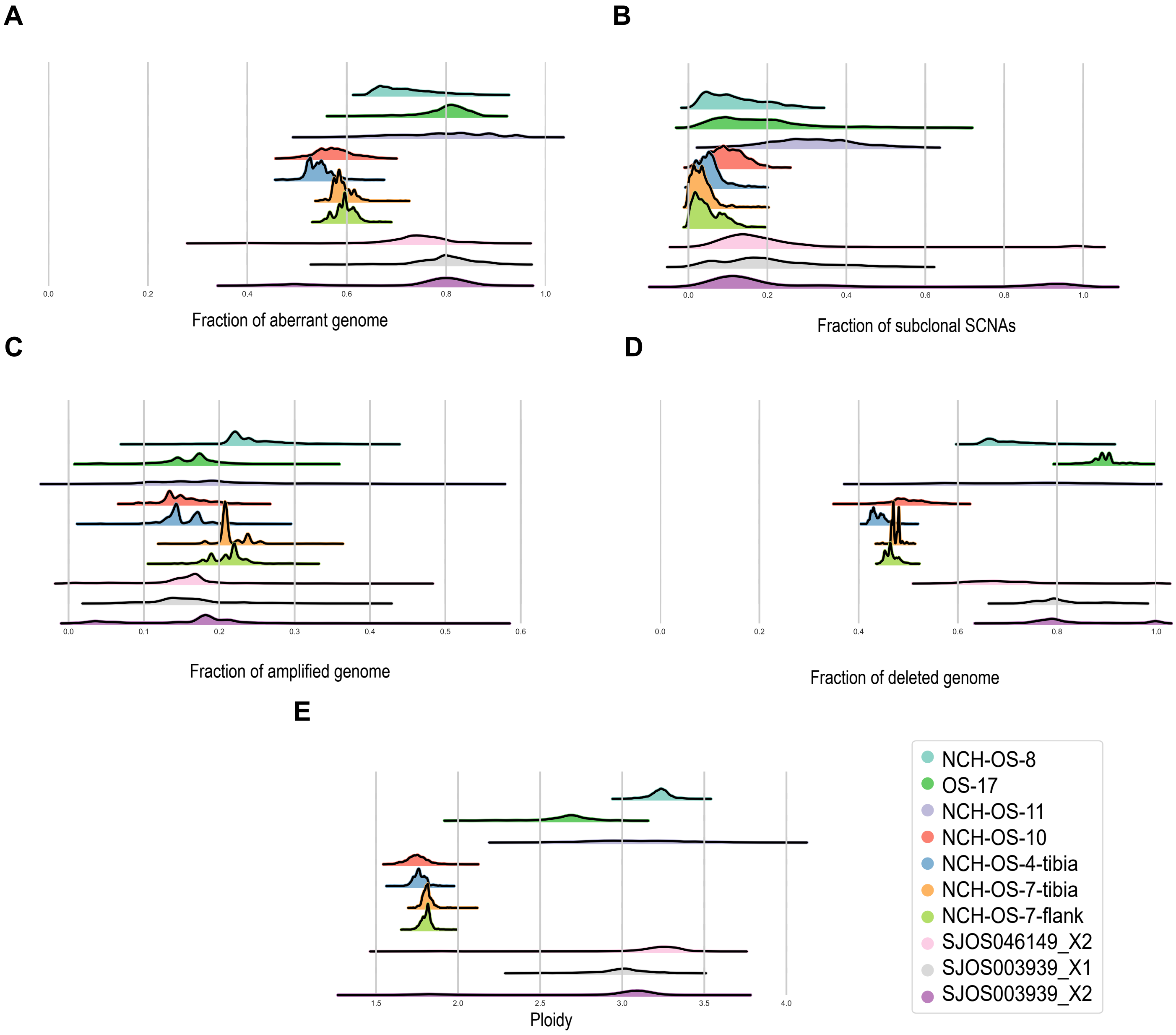

Supplemental Figure S5: Fraction of altered genome density plots. A-E. Ridgeplots show the fraction of the genome that is (A) aberrant, (B) has subclonal variation, exhibits (C) amplification or (D) deletion, and the (E) ploidy of each sample included in the scDNA sequencing dataset.
