# Supplementary material for: Structurally Complex Osteosarcoma Genomes Exhibit Limited Heterogeneity within Individual Tumors and across Evolutionary Time
Source: Cancer Res Commun. 2023 Apr 12;3(4):564–75. doi: 10.1158/2767-9764.CRC-22-0348 (PMC10093779; doi:10.1158/2767-9764.CRC-22-0348)
Supplement: Supplementary Figure S6 — TP53 copy number in single-cell data [file crc-22-0348-s08.pdf]

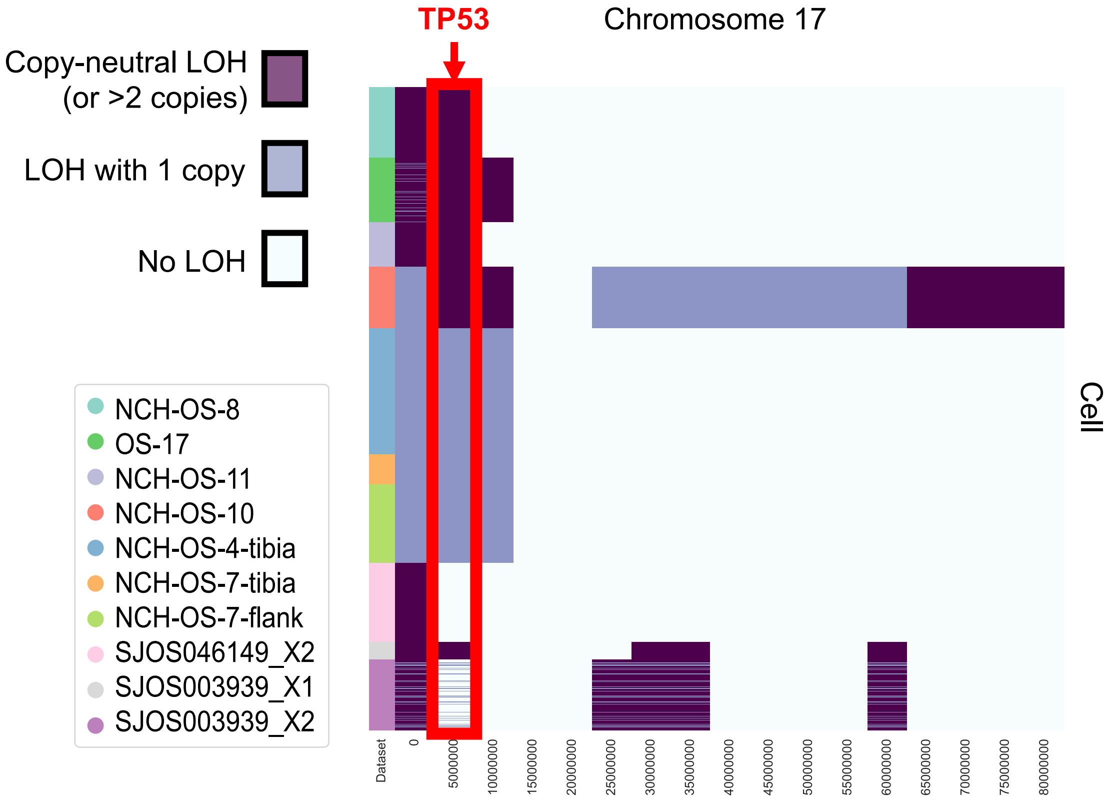

Supplemental Figure S6: TP53 copy number in single-cell data. Copy number status determination for chromosome 17 shows widespread deletion and LOH at the p53 locus cell-by-cell from the scDNA data.
