# Supplementary material for: Structurally Complex Osteosarcoma Genomes Exhibit Limited Heterogeneity within Individual Tumors and across Evolutionary Time
Source: Cancer Res Commun. 2023 Apr 12;3(4):564–75. doi: 10.1158/2767-9764.CRC-22-0348 (PMC10093779; doi:10.1158/2767-9764.CRC-22-0348)
Supplement: Supplementary Figure S8 — Bulk data genome SCNA plots by patient [file crc-22-0348-s10.pdf]

Supplemental Figure 8A

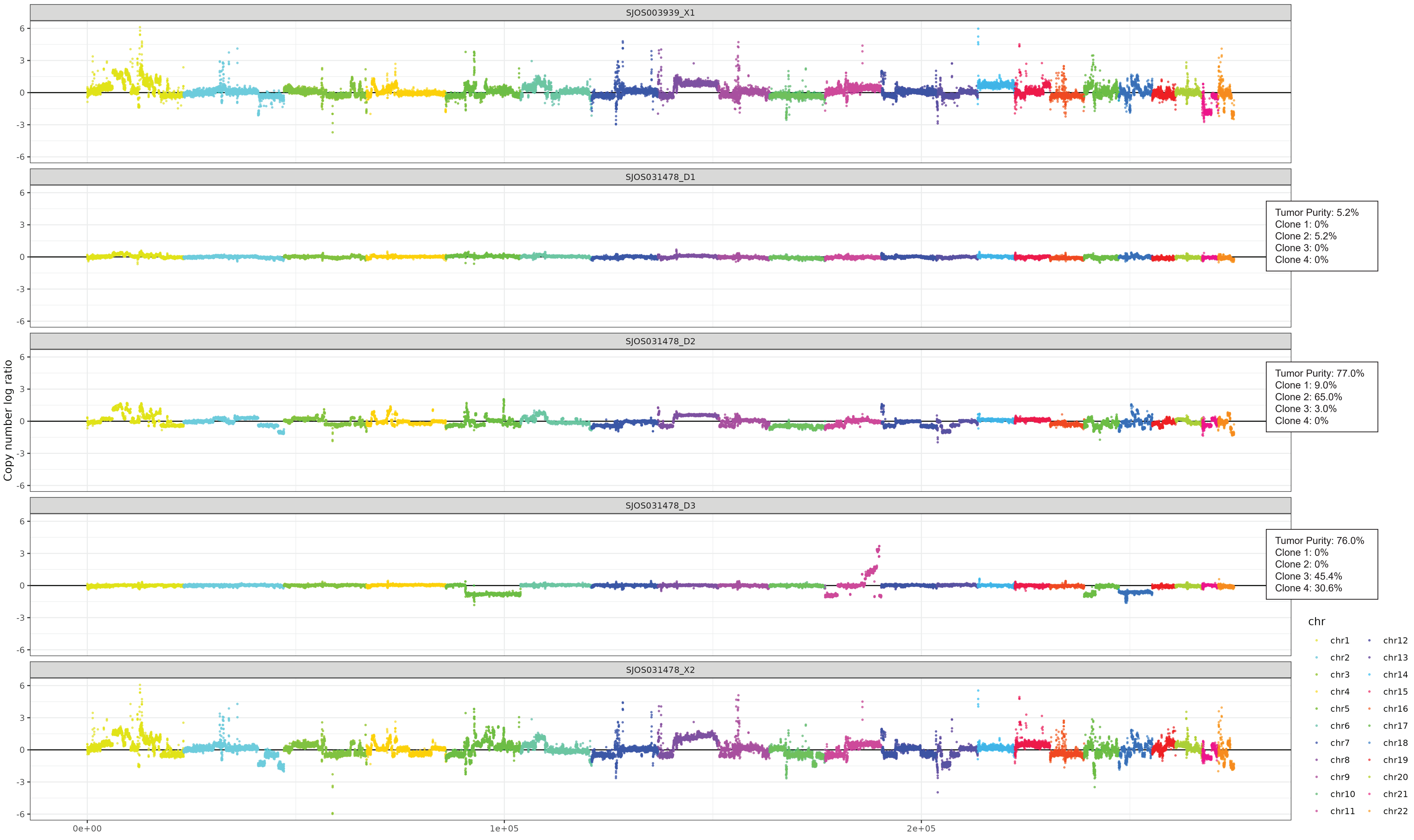

Supplemental Figure 8B

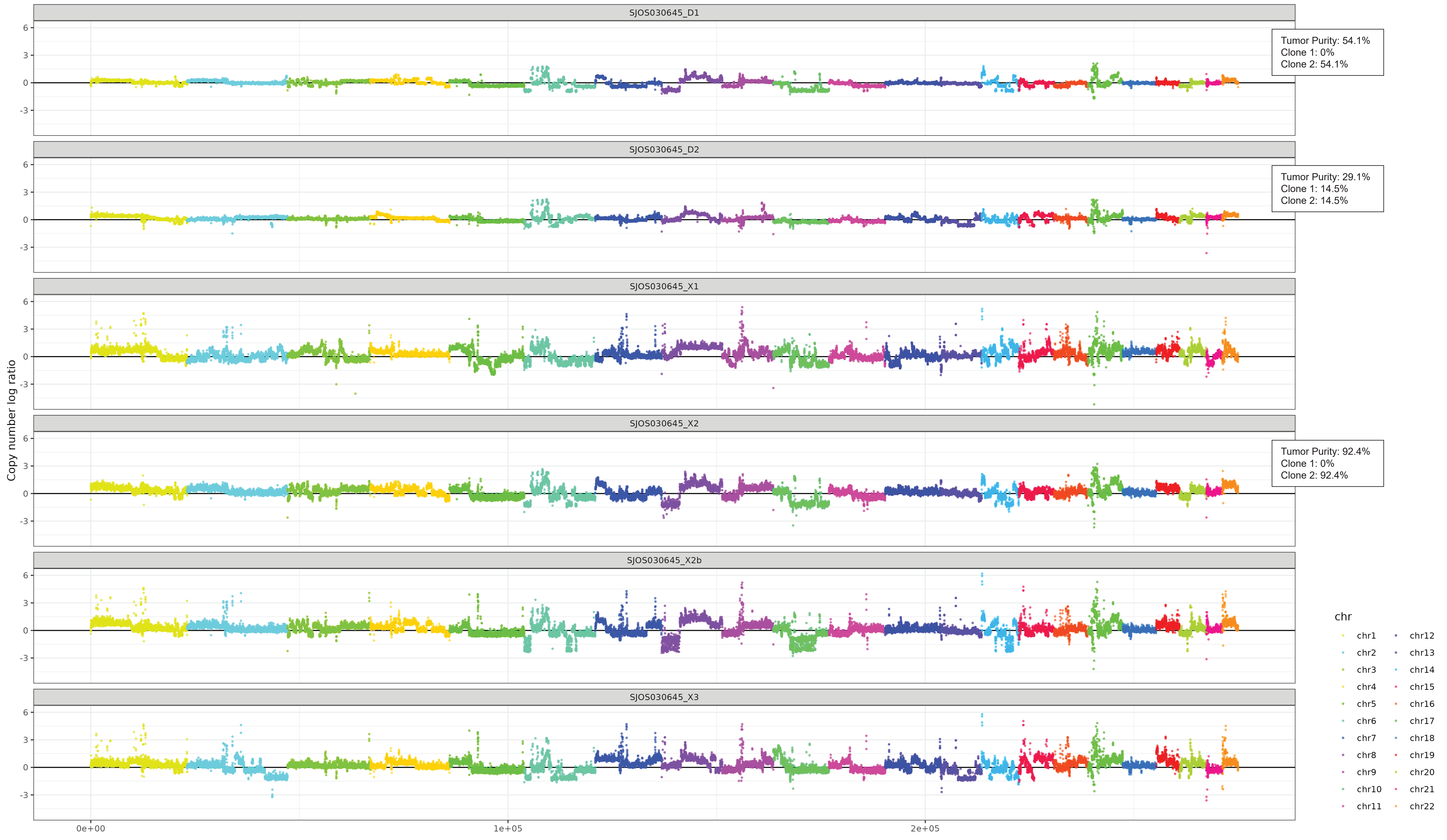

Supplemental Figure 8C

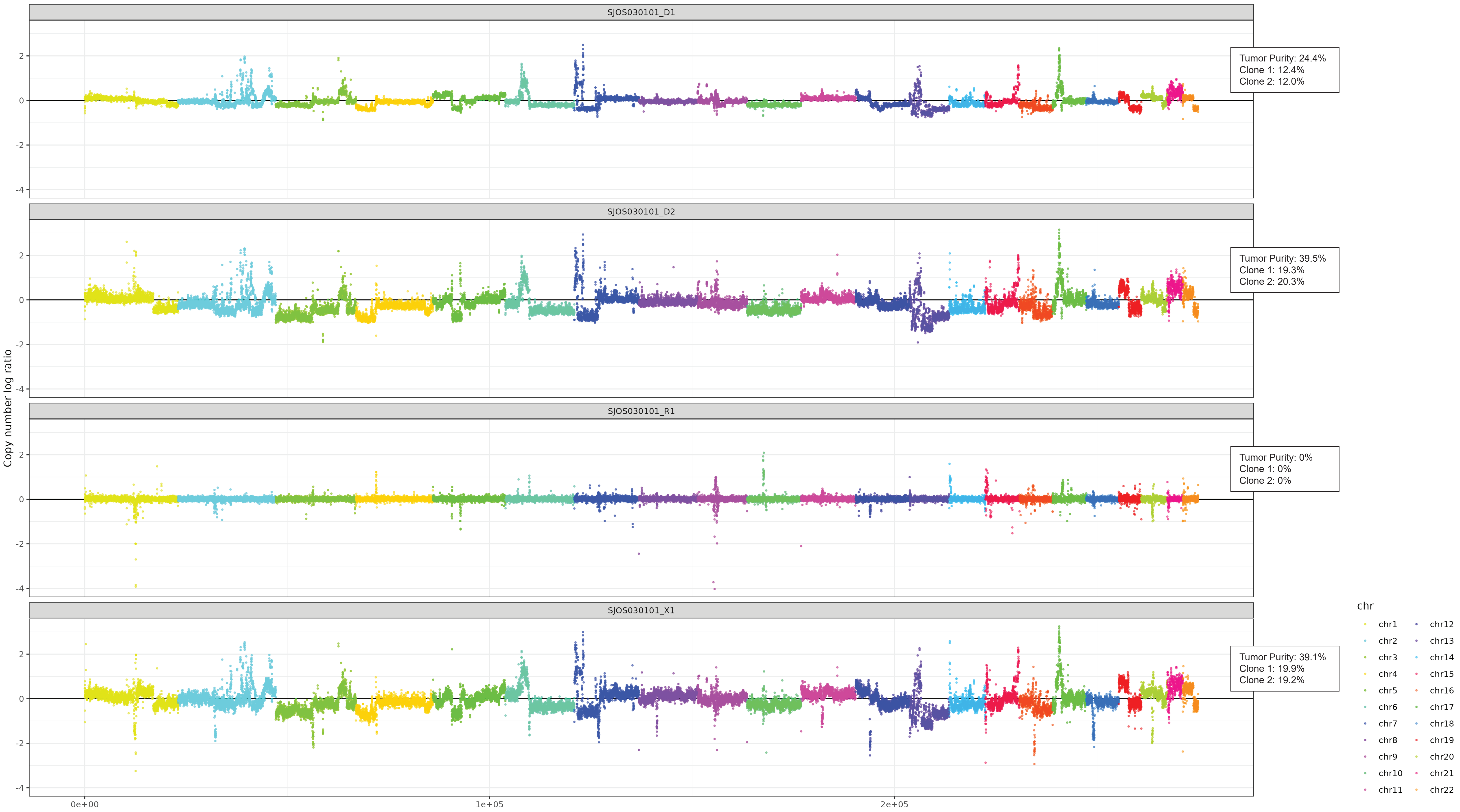

Supplemental Figure 8D

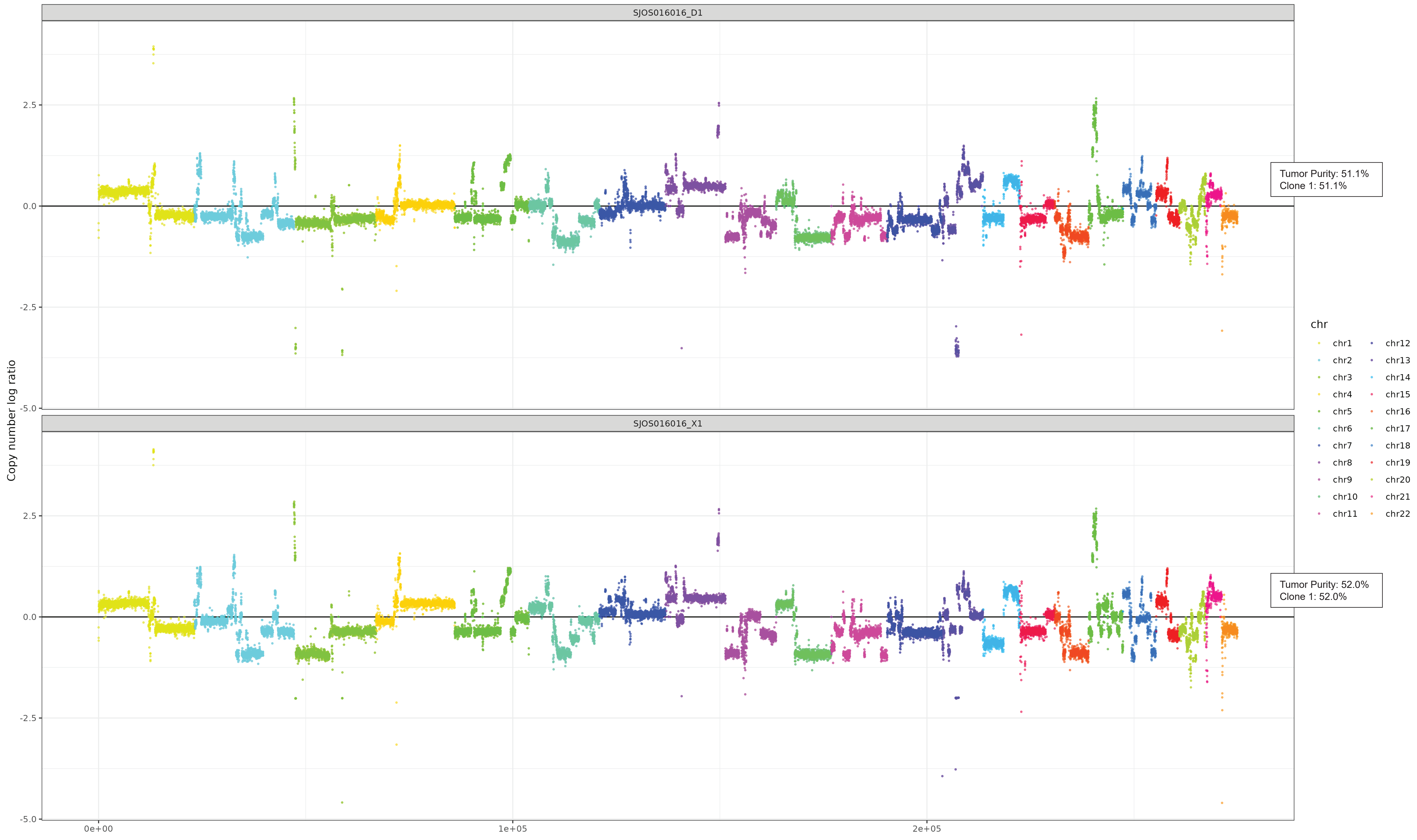

Supplemental Figure 8E

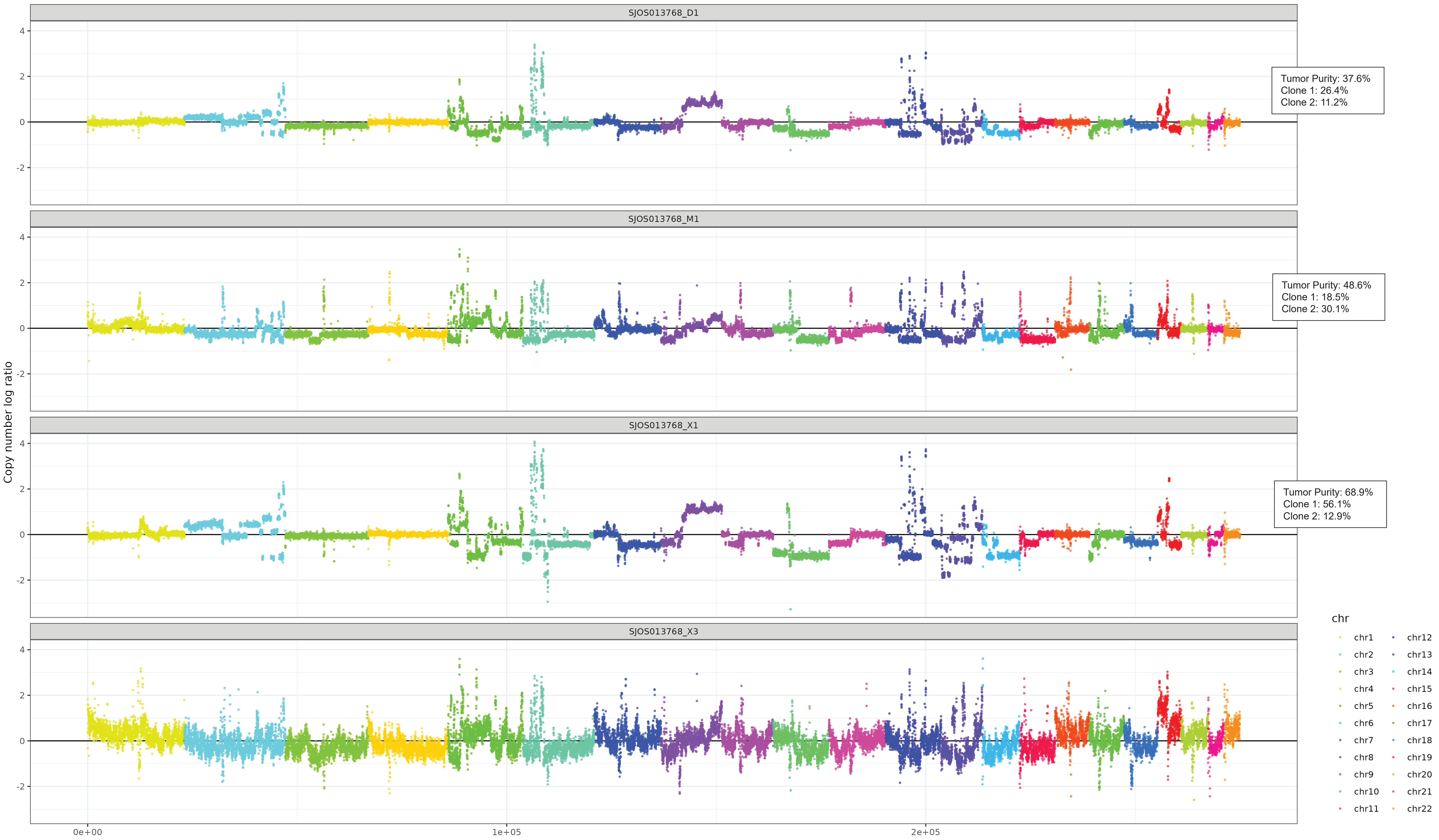

Supplemental Figure 8F

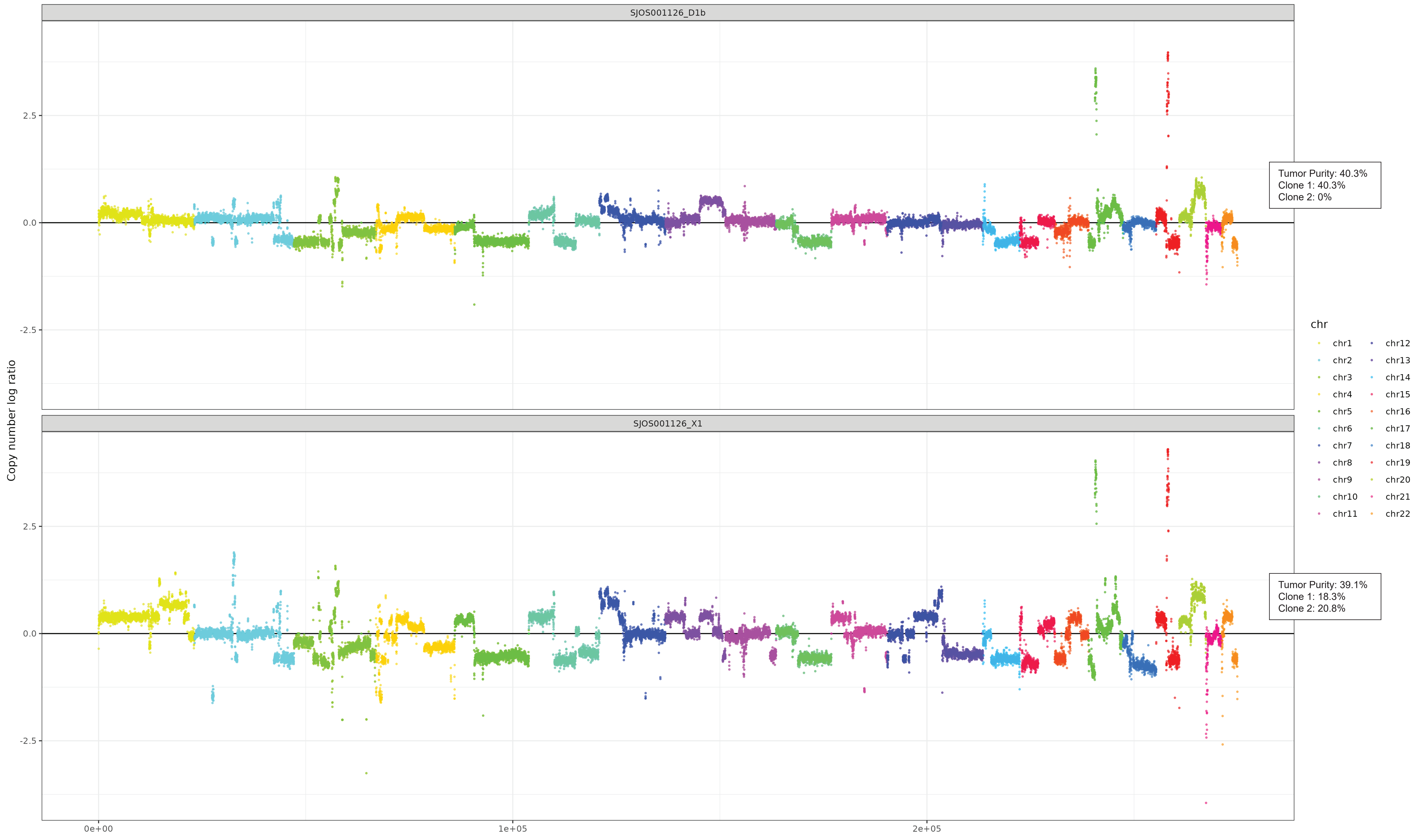

Supplemental Figure 8G

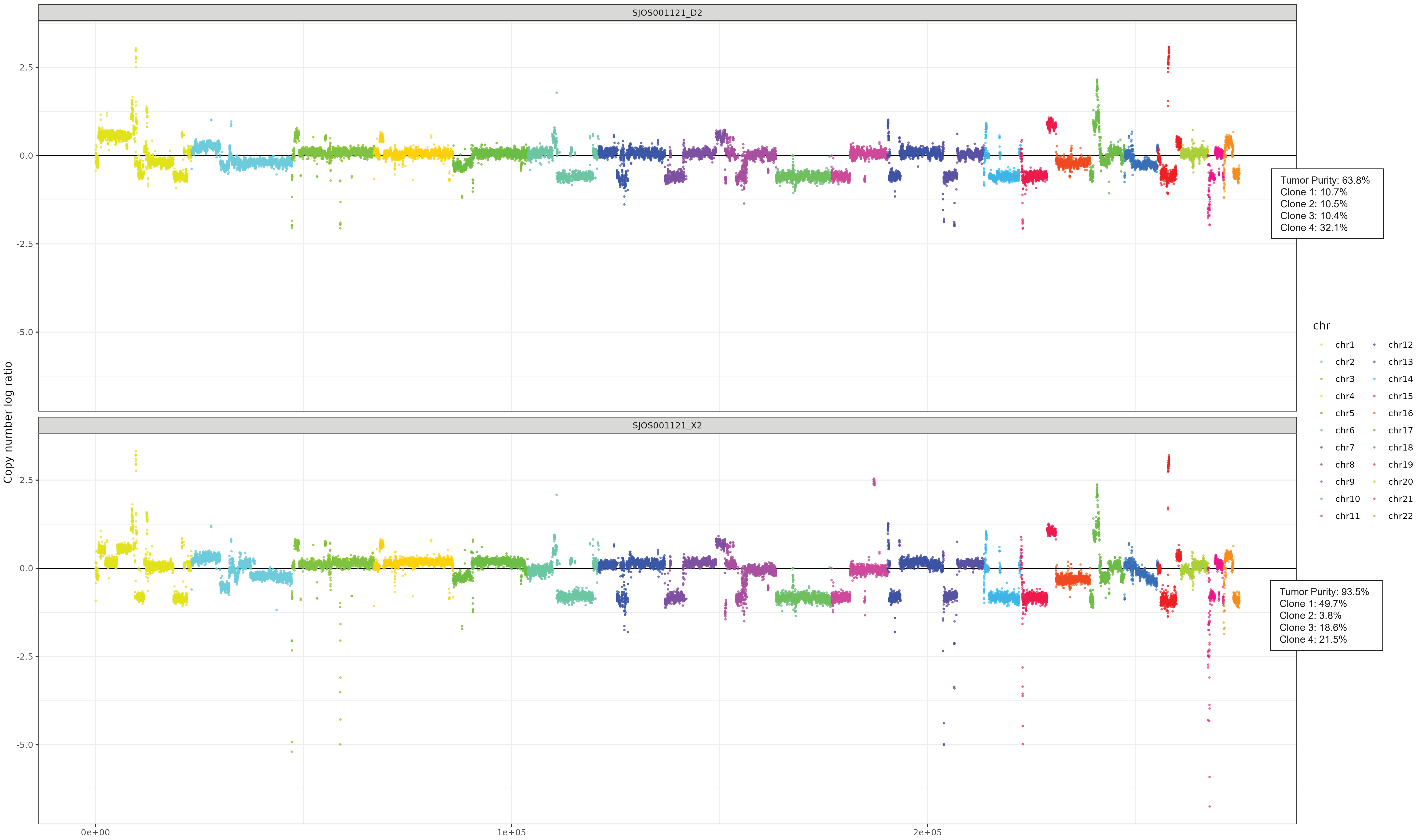

Supplemental Figure 8H

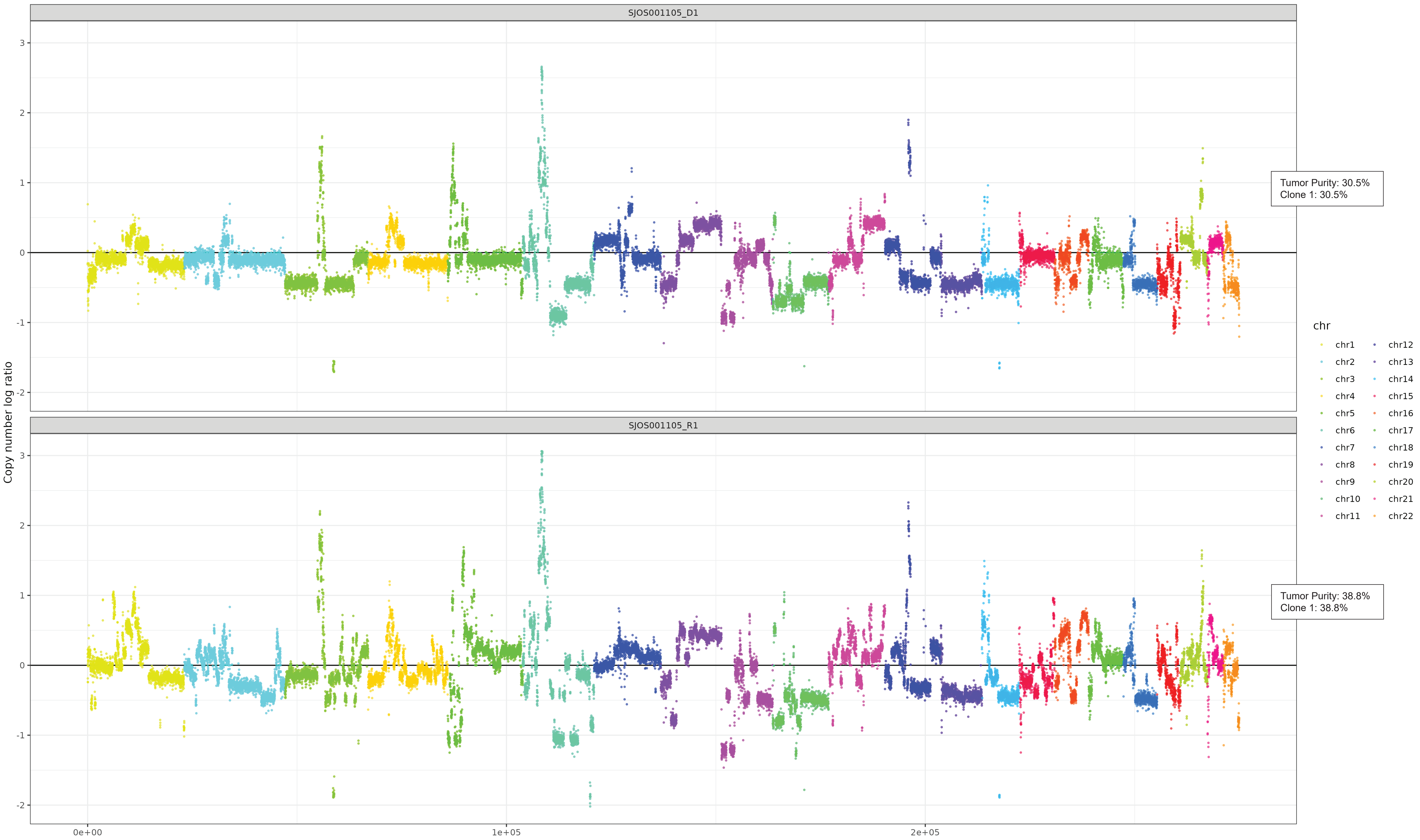

Supplemental Figure 8I

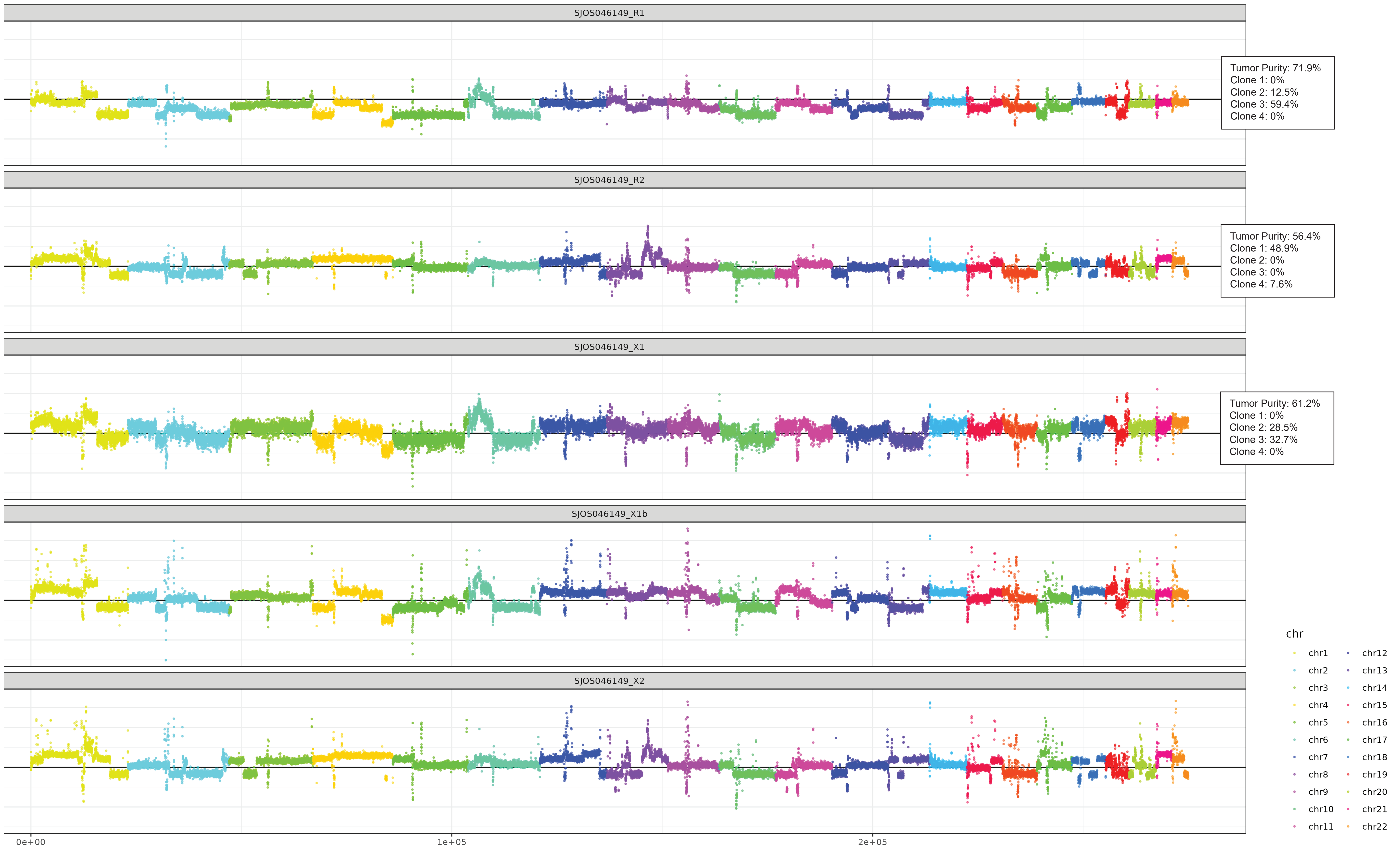

Supplemental Figure 8J

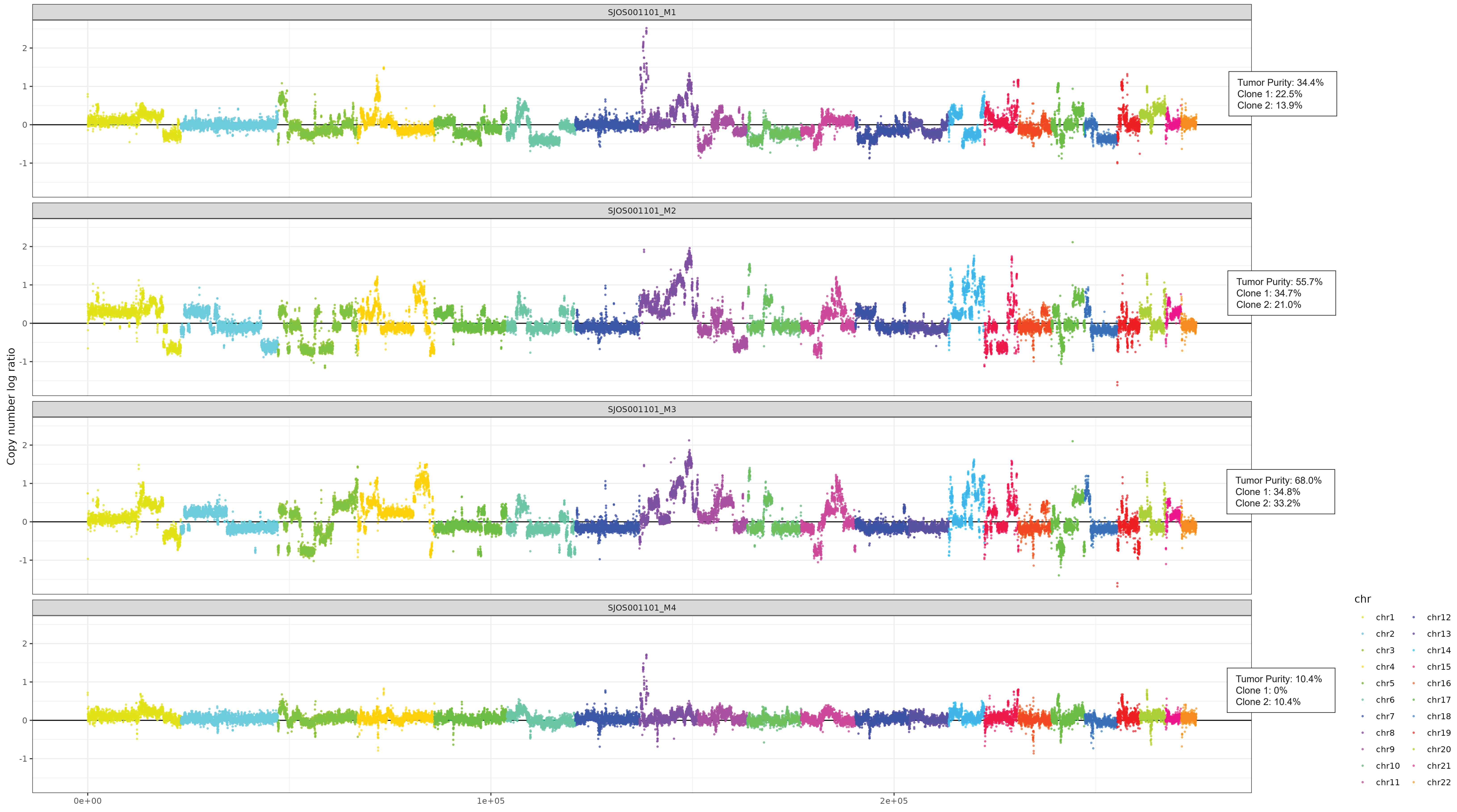

Supplemental Figure S8: St. Jude bulk data genome SCNA plots by patient

A-J. Copy number plots from patients from the St. Jude dataset where WGS data was available from biopsy/resection and/or PDX samples separated by space (metastasis) and/or time. Inset numbers show estimations of tumor purity and SCNA-specific clone composition determined by HATCHET where available.
